# Development and preliminary validation of the post-intensive care syndrome-family assessment scale

**DOI:** 10.3389/fpsyg.2026.1758100

**Published:** 2026-06-24

**Authors:** Wenfan Yu, Zhenzhen Bao, Ruiqin Sha, Yanmin Zheng, Yapeng Tan, Xueyan Guan, Rui Hu, Yaruo Huang, Yonggang Liu, Xueqin Ma, Nianqi Cui, Ying Tian

**Affiliations:** 1 School of Nursing, Kunming Medical University, Kunming, China; 2 Department of Nursing, First Affiliated Hospital of Kunming Medical University, Kunming, China; 3 Yunnan Provincial Psychiatric Hospital, Kunming, China; 4 The Third People's Hospital of Yunnan Province, Kunming, China; 5 Mengzi City People's Hospital, Mengzi, China; 6 Department of Nursing, Yan’an Hospital Affiliated to Kunming Medical University, Kunming, China; 7 Department of Head and Neck Surgery, The Third Affiliated Hospital of Kunming Medical University, Kunming, China

**Keywords:** family caregivers, intensive care unit, PICS-F, post-intensive care syndrome-family, reliability, scale development, validity

## Abstract

**Background:**

Post-Intensive Care Syndrome-Family (PICS-F) is a condition that often develops in family caregivers of ICU patients and severely impacts their quality of life. Currently, there is a lack of comprehensive assessment tools suitable for the Chinese cultural context.

**Objective:**

To develop and validate a culturally appropriate scale for assessing PICS-F in family members of ICU patients within the Chinese context.

**Methods:**

This study adopted an exploratory sequential mixed-methods design. The scale was developed based on a preliminary qualitative study and guided by the Theory of being devastated by the critical illness journey in the family. An initial pool of 42 items was generated. Content validity was evaluated through expert consultation. A pilot survey was conducted. A cross-sectional survey was then performed with 447 family members. Reliability and validity were assessed using item analysis, exploratory factor analysis, confirmatory factor analysis, and Cronbach’s *α* coefficient.

**Results:**

Content validity indices were adequate (item-level content validity index (I-CVI): 0.80–1.00; scale-level content validity index/average (S-CVI/Ave) = 0.98). The final PICS-F Assessment Scale contains 19 items across three dimensions: Psychological Trauma and Distress (8 items), Social and Family Functioning Impairment (8 items), and Deteriorating Physical Health (3 items). Exploratory Factor Analysis (EFA) supported the three-factor structure, accounting for 56.98% of the total variance. Confirmatory Factor Analysis (CFA) indicated adequate model fit [*χ*^2^/d *f* = 2.268, root-mean-square error of approximation (RMSEA) = 0.075, comparative fit index (CFI) = 0.934, Tucker-Lewis index (TLI) = 0.924, Standardized Root Mean Square Residual (SRMR) = 0.064]. The scale demonstrated high internal consistency (Cronbach’s *α* = 0.918 for the total scale; subscales: 0.919, 0.903, 0.851).

**Implications for clinical practice:**

The scale provides a practical tool for early screening and assessment of PICS-F in clinical settings, facilitating timely and targeted support for at-risk family caregivers.

**Conclusion:**

The PICS-F Assessment Scale demonstrated promising preliminary psychometric properties for holistically assessing the impact of critical illness on family members, demonstrating good internal consistency reliability, content validity, and construct validity. Further validation with larger samples and criterion-related measures is needed.

## Introduction

Advances in critical care medicine have improved survival rates for patients admitted to the intensive care unit (ICU) (Schofield-Robinson et al., 2018; Op ‘t Hoog et al., 2020; van Beusekom et al., 2016). Against this backdrop, patient intervention measures centered on early activity and rehabilitation have been proven to significantly improve patient outcomes (Wang et al., 2022; Van Sleeuwen et al., 2020). However, this increasing focus on patient recovery often overlooks the experiences of family members who take on caregiving responsibilities. Family members of ICU patients frequently experience psychological and physical stressors associated with a cluster of symptoms collectively known as Post-Intensive Care Syndrome-Family (PICS-F) (Needham et al., 2012; Davidson et al., 2012; Davidson et al., 2012). PICS-F is defined as new or worsening impairments in psychological, physical, social, and family functioning that emerge after a family member’s ICU admission and may persist beyond hospitalization (Needham et al., 2012). PICS-F manifests as new or worsening impairments in mental health, physical health, social functioning, and family systems (Needham et al., 2012). Health care professionals should be aware of the cultural and spiritual beliefs that influence the perspectives of ICU patients and families, supporting religious beliefs and family in end-of-life care (Charan et al., 2023).

The reported prevalence of PICS-F is high and varies widely, underscoring its significance as a public health concern (Kang, 2023; Petrinec and Martin, 2018). A critical barrier to addressing PICS-F effectively is the absence of a specific, holistic assessment tool. Current research and clinical practice rely on a battery of generic instruments such as the Hospital Anxiety and Depression Scale (HADS) for anxiety/depression (Zigmond and Snaith, 1983), the Impact of Event Scale-Revised (IES-R) for post-traumatic stress disorder (PTSD) (Weiss, 2007) to measure isolated symptoms. This method increases the difficulty of integrating existing PICS-F-related research (Love Rhoads et al., 2022) and is not conducive to evidence synthesis or subsequent evidence-based practice and intervention. Hayes et al. (2024) highlighted the lack of comprehensive tools for PICS-F assessment, while Wen et al. (2025) further supported the multidimensional conceptual framework of PICS-F.

The necessity for a culturally sensitive instrument is particularly salient in the Chinese context, where family dynamics and caregiving experiences are shaped by distinct sociocultural values (Liu et al., 2022; Jiang et al., 2025; Peng et al., 2022). First, rooted in Confucian collectivism and the cardinal virtue of xiao (filial piety), the family serves as the central locus of decision-making in Chinese ICUs, where critical care choices are typically made through family consensus rather than individual patient autonomy (Liu et al., 2022). Second, Chinese family caregivers confront unique stressors that intertwine economic and moral dimensions: substantial out-of-pocket medical expenses coexist with a profound sense of moral obligation to “do everything possible” creating a dual burden of financial strain and treatment-related guilt that may exceed Western experiences (Li et al., 2023; Tang et al., 2022). Third, these cultural specificities suggest that the manifestation of PICS-F in Chinese populations may differ qualitatively from Western cohorts, potentially featuring more pronounced guilt-related symptoms and family-centered psychological distress rather than individualistic trauma responses (Kim et al., 2008; Ryder et al., 2012; Maercker et al., 2009). Consequently, direct application of Western-derived PICS-F assessment tools to Chinese family caregivers risks overlooking culture-specific symptom manifestations and may yield incomplete or biased evaluations of post-ICU family outcomes. Therefore, developing a robust and culturally appropriate scale is essential to accurately assess PICS-F among family caregivers in China.

With this in mind, the present study is guided by Kang (2023) “Theory of Being Devastated by the Critical Illness Journey in the Family,” a constructivist grounded theory. Kang (2023) theory was developed through constructivist grounded theory interviews with 8 ICU family members. The core category, “being devastated,” reflects the profound and multidimensional disruption that critical illness imposes on family caregivers. According to this theory, PICS-F is not merely a set of isolated symptoms but a “devastated life” comprising four interconnected domains: Psychological Trauma and Distress (e.g., anxiety, depression, helplessness, guilt), Deteriorating Physical Health (e.g., sleep disturbance, fatigue, weight loss), Social Withdrawal (e.g., reduced work efficiency, loss of social activities), and Family Crisis (e.g., financial strain, reduced family communication, role conflicts) (Kang, 2023). This theoretical framework was further validated and refined through our own qualitative study (Bao et al., under review) conducted with 18 Chinese ICU family members, which confirmed the relevance of these four domains in the Chinese cultural context. Based on this cultural grounded theoretical framework, we generated an initial item pool covering all four domains. Subsequent exploratory factor analysis, however, empirically merged Social Withdrawal and Family Crisis into a single dimension (Social and Family Functioning Impairment), resulting in the final three-factor structure of the scale (Psychological Trauma and Distress, Social and Family Functioning Impairment, and Deteriorating Physical Health). This theory therefore provides the conceptual foundation for item generation and directly informs the scale’s dimensionality.” The present study therefore aimed to develop and preliminarily validate a culturally sensitive scale for assessing PICS-F in Chinese family caregivers of ICU patients.

## Methods

### Study design

**Figure 1:**
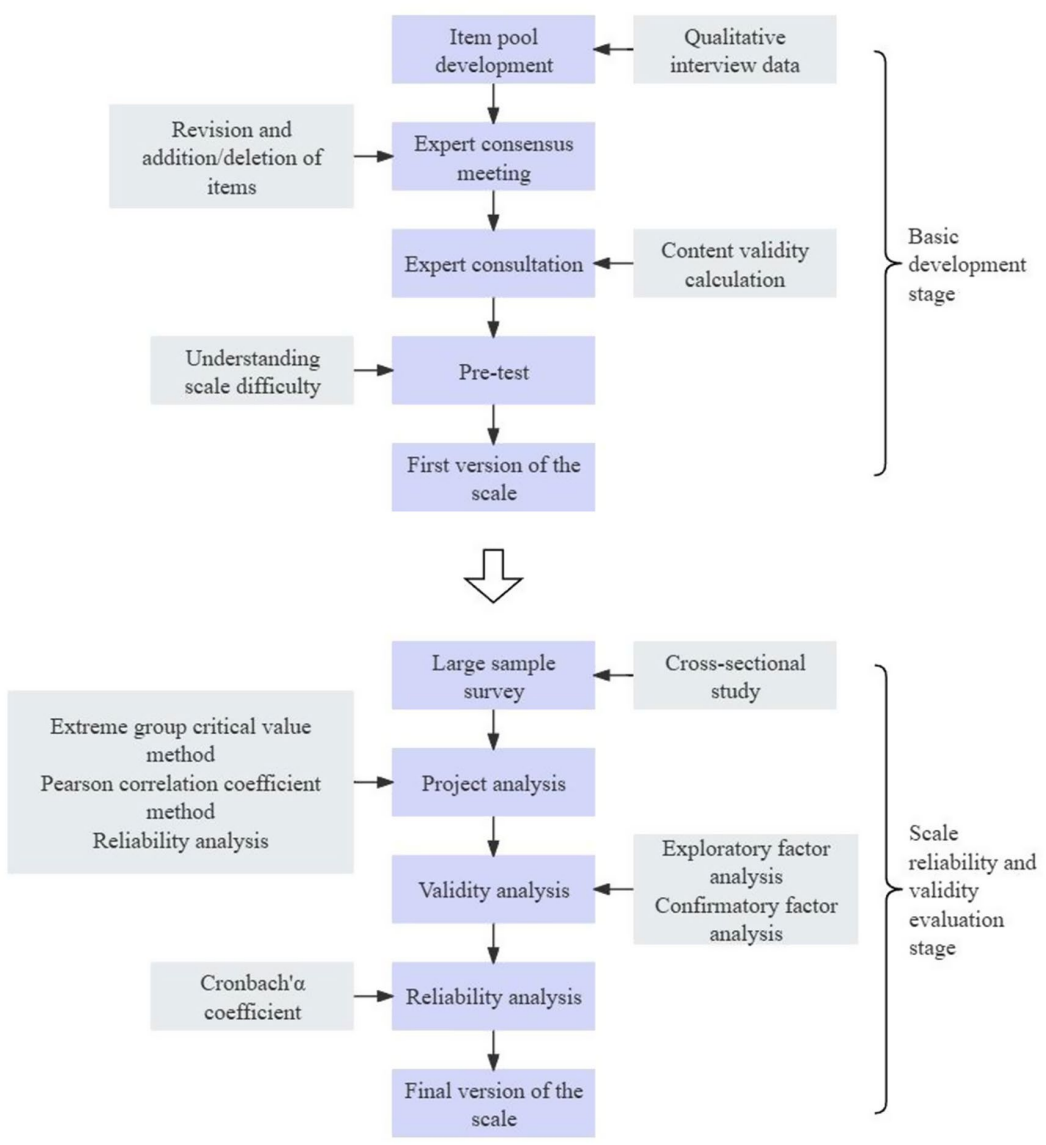
Flowchart of the scale development and validation process.

This study followed established psychometric guidelines (Streiner et al., 2015) to develop the PICS-F scale, with its conceptual foundation informed by the “Theory of being devastated by the critical illness journey in the family” (Kang, 2023). An exploratory sequential mixed-methods design was adopted to integrate qualitative and quantitative approaches. The development process consisted of three main phases: (1) item generation through a preliminary qualitative study and content validity assessment; (2) pilot testing; and (3) psychometric validation (Figure 1).

### Item generation and content validity

#### Item pool construction

The initial item pool was generated based on the four-domain conceptual framework derived from Kang (2023) theory, which was operationalized and contextualized through our preliminary qualitative study (Bao et al., under review), conducted from June to September 2023 using purposive sampling with 18 Chinese ICU family members. Verbatim expressions and thematic content from the interview transcripts were transformed into a preliminary set of 42 items (Appendix 1) across the four dimensions: Psychological Trauma and Distress (25 items), Deteriorating Physical Health (6 items), Social Withdrawal (5 items), and Family Crisis (6 items). Items were phrased as first-person statements rated on a Likert scale.

#### Expert consensus

An expert panel of eight professionals was convened. The experts were from critical care medicine, psychiatry, critical care nursing, psychological nursing, and nursing management, with 5–26 years of professional experience. The panel included a clinical psychologist, who participated in both the expert consensus meeting and the subsequent expert consultation. They evaluated the items for representativeness, clarity, comprehensiveness, and appropriateness. Based on their feedback, items were merged, reworded, added, or deleted, resulting in a refined 40-item pool (Appendix 2).

The expert consensus meeting consisted of eight members, including two investigators (Ma XQ and Liu YG) and six external experts. Prior to the meeting, all participants were informed of the investigators’ role, and their participation was confirmed after obtaining consent from all experts. According to the ICMJE authorship criteria (Yadav, 2024; International Committee of Medical Journal Editors, 2024; COPE Council, 2019), the two investigators met all four criteria for authorship and are listed as co-authors. The six external experts from the consensus meeting and the 10 experts from the expert consultation panel were external contributors and are acknowledged.

#### Expert consultation

A subsequent expert consultation was conducted with a separate panel of 10 experts from four provinces. All experts held doctoral or master’s degrees and had 13–30 years of experience across critical care nursing, clinical psychological nursing, nursing management, critical care medicine, and nursing education. Experts rated each item on its relevance to the corresponding dimension using a 4-point Likert scale (1 = not relevant, 4 = highly relevant). The Item-Level Content Validity Index (I-CVI), Scale-Level Content Validity Index/Average (S-CVI/Ave), and Scale-Level Content Validity Index/Universal Agreement (S-CVI/UA) were calculated. Items with I-CVI < 0.78 were considered for deletion (Polit and Beck, 2006). This process yielded adequate content validity indices (I-CVI = 0.80–1.00, S-CVI/Ave = 0.98, S-CVI/UA = 0.85), leading to the deletion of one item and modification of 11 others, resulting in a 39-item version (Appendix 3).

#### Pilot testing

The pilot test was conducted in October 2023 using purposive sampling with 20 family members whose demographic characteristics are summarized in Table 1. After completing the 39-item preliminary scale, comprehension, clarity, and feasibility were assessed through oral interview. All 20 participants reported that the items were understandable and clearly worded. Feasibility was defined as the ability to complete the scale independently within a reasonable time (15 min or less) (Eldridge et al., 2016; Willis, 2005). The mean completion time was 8 min (range 5–15 min). No item modifications were needed, resulting in the first version of the PICS-F Assessment Scale with 39 items.

**Table 1:** Demographic characteristics of the 20 pilot participants.

ID	Sex	Relationship to patient	Patient still in ICU	Ethnicity	Insurance type
F1	M	Son	Yes	Han	Provincial Cross-Regional Basic Medical Insurance
F2	M	Father	Yes	Yi	Self-Pay
F3	M	Son	Yes	Han	Self-Pay
F4	F	Daughter	Yes	Han	Provincial-level Urban Employee Basic Medical Insurance
F5	F	Wife	Yes	Han	Self-Pay
F6	F	Daughter	Yes	Han	Provincial Cross-Regional Basic Medical Insurance
F7	M	Husband	Yes	Han	Provincial Cross-Regional Basic Medical Insurance
F8	M	Father	Yes	Han	Provincial Cross-Regional Basic Medical Insurance
F9	F	Mother	Yes	Han	Provincial Cross-Regional Basic Medical Insurance
F10	F	Daughter	Yes	Lahu	Provincial Cross-Regional Basic Medical Insurance
F11	F	Wife	Yes	Han	Self-Pay
F12	M	Husband	Yes	Han	Provincial Cross-Regional Basic Medical Insurance
F13	F	Daughter	Yes	Han	Provincial Cross-Regional Basic Medical Insurance
F14	F	Mother	Yes	Hui	Provincial Cross-Regional Basic Medical Insurance
F15	F	Sister	Yes	Bulang	Provincial-level Student Basic Medical Insurance
F16	F	Wife	Yes	Han	Provincial Cross-Regional Basic Medical Insurance
F17	M	Son	Yes	Han	Provincial Cross-Regional Basic Medical Insurance
F18	M	Son	Yes	Han	Provincial Cross-Regional Basic Medical Insurance
F19	F	Wife	Yes	Han	Provincial Portable Basic Medical Insurance for Employees
F20	M	Son	Yes	Han	Provincial Portable Basic Medical Insurance for Employees

Age was not systematically recorded during the pilot phase; participants’ ages were observed to range approximately from 25 to 65 years.

### Psychometric validation

#### Participants and setting

From November to December 2023, questionnaires were distributed to the families of patients in various ICUs and patients transferred from ICUs to general wards in a certain tertiary grade-A hospital in Yunnan Province using the convenience sampling method. The study was conducted in multiple ICUs at this tertiary teaching hospital in China. These ICUs primarily admit patients with critical illnesses, including severe sepsis, multi-organ dysfunction, post-cardiac arrest, and major trauma. The majority of patients require mechanical ventilation and vasopressor support, reflecting a high baseline illness severity. While individual-level severity scores were not routinely documented during the study period, the overall patient population can be characterized as severely ill based on admission diagnoses and treatment intensity.

Eligible participants were family members of patients aged 18 years or older who had been admitted to the ICU for at least 24 h and were either currently in the ICU or had been transferred to a general ward. Family members were required to be at least 18 years of age and have a relationship to the patient limited to parents, spouses, children, or siblings. All participants provided written informed consent prior to enrollment. Family members were excluded if they had a previous history of mental illness, severe psychological or cognitive dysfunction, or had experienced other severe traumatic stress events within the past 3 months. Participants who withdrew during the study were also excluded from the final analysis. A total of 447 valid responses were included in the final analysis.

Of the 550 distributed questionnaires, 447 were valid. A total of 103 participants were excluded: 54 did not return the questionnaire, and 49 had incomplete or patterned responses.

#### Sample size

The target sample size was determined to be 5–10 times the number of items (Osborne and Costello, 2004), accounting for a potential 10–20% invalid response rate. With 39 items, a sample of 215–468 was required. We ultimately collected 447 valid responses. The dataset was randomly split into two halves for subsequent psychometric validation: one half for exploratory factor analysis (EFA, *n =* 223) and the other half for confirmatory factor analysis (CFA, *n =* 224).

#### Data collection

Eligible participants completed a questionnaire package containing a demographic information sheet and the 39-item preliminary PICS-F scale. The scale used a 5-point Likert response format (1 = strongly disagree to 5 = strongly agree), with higher scores indicating more severe PICS-F symptoms.

#### Data analysis

Data were analyzed using SPSS Statistics for Windows, Version 26.0 (IBM Corp., Armonk, NY, USA) and AMOS for Windows, Version 26.0 (IBM Corp., Armonk, NY, USA).

Item analysis was employed on the total sample (*N* = 447) to evaluate the discriminative power of each item. The critical ratio (CR) method was used, retaining items with CR > 3.0.

#### Construct validity

Exploratory Factor Analysis (EFA): Principal axis factoring with direct oblique rotation was performed on the first half of the sample (*n =* 223). Oblique rotation was chosen because factors were expected to be correlated (Fabrigar et al., 1999). The number of factors was determined by eigenvalues >1, scree plot inspection, and conceptual interpretability. In each EFA iteration, we evaluated factor loadings and cross-loadings. A “cross-loading” was defined as an item having a standardized factor loading of 0.32 or higher on two or more factors (Osborne and Costello, 2009). Items with a primary factor loading below 0.45 (Comrey and Lee, 1992) or with cross-loadings ≥ 0.32 were removed. Additionally, an item was considered for removal if it did not load meaningfully on its theoretically expected factor based on the pattern matrix and conceptual interpretability (Worthington and Whittaker, 2006). Items were removed one at a time, and EFA was re-run after each removal (23 rounds in total), following established scale development practices (Worthington and Whittaker, 2006). A total of 23 iterative rounds were conducted, applying these pre-specified statistical criteria together with theoretical interpretability to guide item deletion.

Confirmatory Factor Analysis (CFA): Maximum likelihood estimation was used on the second half of the sample (*n =* 224) to test the model fit derived from EFA. Model fit was assessed using a combination of widely recommended indices (Hu and Bentler, 1999; Kline, 2011): the chi-square-to-degrees-of-freedom ratio (*χ*^2^/df), the root-mean-square error of approximation (RMSEA), the comparative fit index (CFI), the Tucker-Lewis index (TLI), and the standardized root-mean-square residual (SRMR). Following established guidelines, a *χ*^2^/df value < 3.0 indicated acceptable fit, while RMSEA < 0.08, CFI > 0.90, TLI > 0.90, and SRMR < 0.08 were considered indicators of adequate model fit (Hu and Bentler, 1999). Modification indices (MI) were examined to identify local misfit. Only modifications with MI > 10 were considered. Any added covariance between error terms required a theoretical or content-based justification. The expected parameter change (EPC) was also examined to ensure the substantive meaningfulness of the suggested modification. Convergent validity was assessed using Average Variance Extracted (AVE > 0.5) and Composite Reliability (CR > 0.7) as suggested by Fornell and Larcker (1981). Discriminant validity was confirmed if the square root of the AVE for each factor was greater than the factor’s correlations with other factors.

#### Reliability

Internal consistency was evaluated using Cronbach’s coefficient for the total scale and each subscale. Following the criteria recommended by DeVellis (2003) and George and Mallery (2003), Cronbach’s *α* ≥ 0.90 was considered excellent, 0.80 ≤ *α* < 0.90 good, 0.70 ≤ *α* < 0.80 acceptable, and *α* < 0.70 questionable.

#### Ethical consideration

The study received formal ethical approval from the Hospital Ethics Committee (Approval no: [2022] Lun Shen L 293). All potential participants were approached by a trained research nurse in a private room within the ICU waiting area or general ward. The researcher explained the purpose, procedures, potential risks, and benefits of the study in detail, and participants were given sufficient time to ask questions. Written informed consent was obtained from each participant prior to data collection. Participants were assured that their participation was entirely voluntary and that they could withdraw at any time without affecting the patient’s care or their relationship with the hospital. All data were kept strictly confidential and used solely for the purposes of this study.

## Results

### Participants characteristics

A total of 447 family members completed the survey. The majority were female (53.69%), children of the patients (55.93%), and had a partial or full understanding of the patient’s condition (94.13%). The mean time from ICU admission to questionnaire completion was 7 days (media*n =* 4, range = 1–61). Detailed demographic characteristics of the participants and their respective patients are presented in Table 2.

**Table 2:** Demographic Characteristics of Family Members and Patients (n=447)

**Characteristics**	**Categories**	**n(%)/** **Mean±SD**
**Family caregivers**		
Relationship to the patient	Parent	30(6.71)
	Spouse	121(27.07)
	Sibling	46(10.29)
	Child	250(55.93)
Sex	Male	207(46.31)
	Female	240(53.69)
Age (years)	18-30	76(17.00)
	31-45	173(38.70)
	46-60	136(30.43)
	>60	62(13.87)
Ethnicity	Han	352(78.75)
	Ethnic Minority	95(21.25)
Educational level	Primary school or below	51(11.41)
	Junior high school	103(23.04)
	High school or vocational school	96(21.48)
	College or Bachelor’s degree	184(41.16)
	Master’s degree or above	13(2.91)
Occupation	Government/Institutional employee	60(13.42)
	Corporate employee	87(19.46)
	Self-employed/Business	37(8.28)
	Farmer	94(21.03)
	Freelancer	82(18.35)
	Unemployed	22(4.92)
	Retired	49(10.96)
	Student	16(3.58)
Household monthly income (CNY)	≤3000	93(20.80)
	3001-6000	154(34.45)
	6001-9000	97(21.70)
	>9000	72(16.11)
	Unknown	31(6.94)
Knowledge about patient's illness	Very poor	13(2.91)
	Poor	12(2.69)
	Moderate	265(59.28)
	Good	157(35.12)
**Patients**		
Sex	Male	286(63.98)
	Female	161(36.02)
Age(years)	18-40	76(17.00)
	41-60	168(37.58)
	61-80	166(37.14)
	>80	37(8.28)
ICU Length of Stay(days)	1-2	149(33.33)
	3-5	122(27.30)
	6-10	87(19.46)
	>10	89(19.91)
Days after ICU Discharge(days)	0(Day of discharge)	344(76.96)
	1-2	64(14.32)
	3-5	28(6.26)
	>5	11(2.46)
Route of ICU Admission	Emergency Department	172(38.48)
	General Ward	47(10.51)
	Post-operation	96(21.48)
	Transfer from another hospital	132(29.53)
Medical Payment Type	Resident Basic Medical Insurance	232(51.90)
	Employee Basic Medical Insurance	167(37.36)
	Out-of-pocket	43(9.62)
	Medical Assistance for low-income families	2(0.45)
	Provincial Insurance for Veterans	3(0.67)

### Item analysis

All 39 initial items met the criteria for retention. The CR values ranged from 7.888 to 28.822 (all *p* < 0.001).

### Construct validity

#### Exploratory factor analysis (EFA)

The Kaiser-Meyer-Olkin (KMO) value of this study ranged from 0.900 to 0.935 (>0.800) and Barlett’s test of sphericity was significant (*P* < 0.05), indicating that it is suitable for exploratory factor analysis.

**Figure 2:**
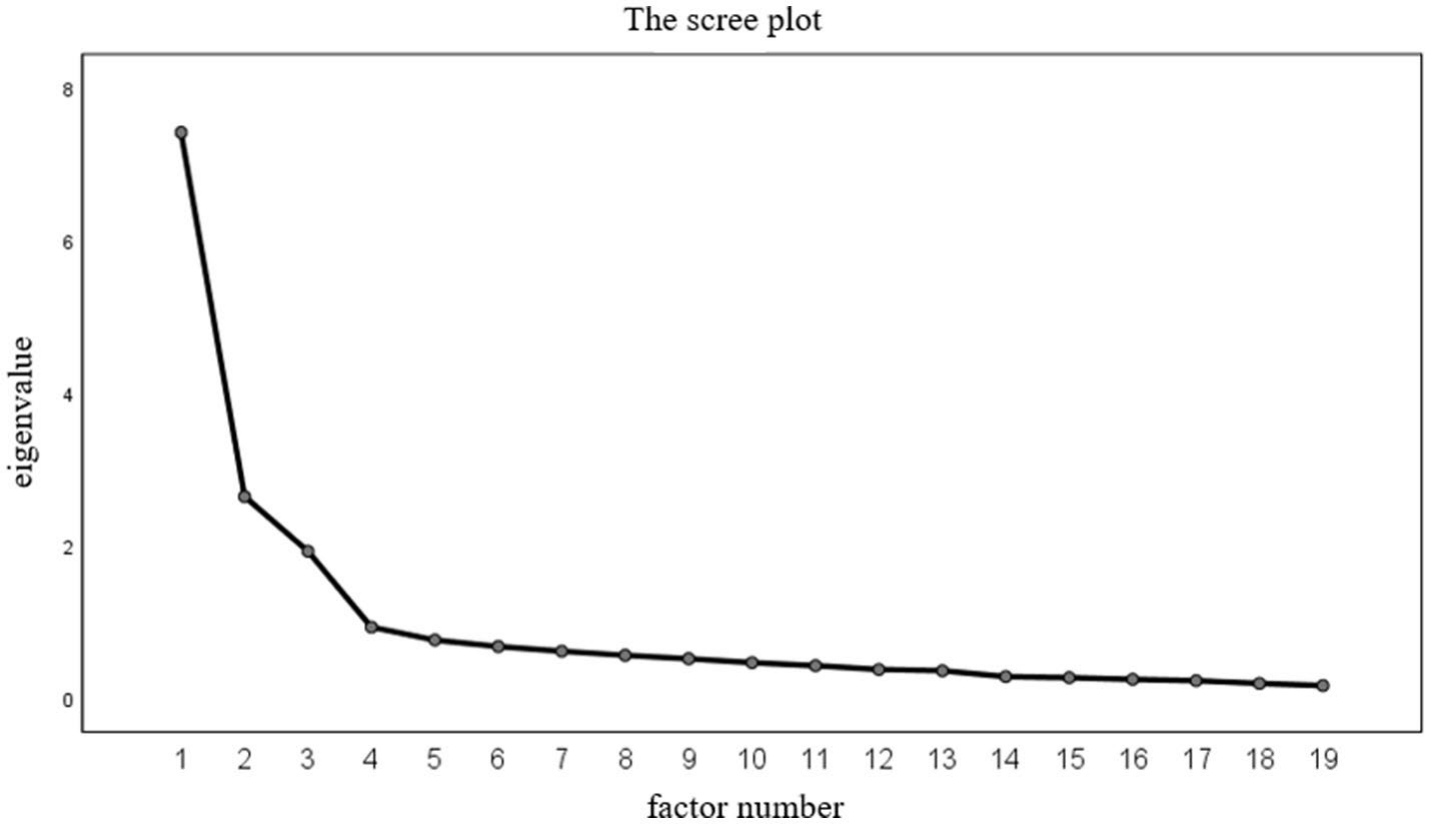
The scree plot of exploratory factor analysis.

Although Kang (2023) theory originally proposed four domains (Psychological Trauma and Distress, Deteriorating Physical Health, Social Withdrawal, and Family Crisis), the EFA results empirically merged Social Withdrawal and Family Crisis into a single factor. After 23 iterative rounds of item-by-item deletion (each round: remove items with primary loading <0.45 (Comrey and Lee, 1992) or cross-loading ≥0.32, or items that did not align with the theoretically expected factor), 20 items were removed, and the final three-factor 19-item solution was obtained. All deletion criteria were pre-specified (Osborne and Costello, 2009; Hair et al., 1998). The final EFA on the remaining 19 items revealed a clear three-factor structure (Table 3). The factors were labeled as: Factor 1: Psychological Trauma and Distress, Factor 2: Social and Family Functioning Impairment (merged from Social Withdrawal and Family Crisis), Factor 3: Deteriorating Physical Health, The scree plot is shown in Figure 2.

**Table 3:** Factor loadings from the exploratory factor analysis of the PICS-F Scale (*n =* 223).

Item	Factor loading		
	F1	F2	F3
C5	0.831	0.072	0.011
C6	0.795	0.038	0.084
C3	0.777	0.052	0.007
C1	0.748	0.032	−0.042
C4	0.730	−0.090	0.053
C2	0.703	−0.086	−0.089
C8	0.622	0.166	0.096
C10	0.509	0.064	0.109
C31	−0.100	0.887	−0.070
C30	−0.028	0.817	−0.031
C37	−0.125	0.717	0.056
C34	0.184	0.659	0.039
C35	0.150	0.611	0.098
C32	0.143	0.599	−0.113
C13	0.150	0.590	−0.021
C28	−0.052	0.569	0.150
C25	0.004	−0.027	0.887
C23	0.029	−0.079	0.857
C24	0.074	0.184	0.719
Variance explained (%)	36.84	11.59	8.55
Cumulative variance (%)	36.84	48.43	56.98

F1, Psychological Trauma and Distress (8 items); F2, Social and Family Functioning Impairment (8 items); F3, Deteriorating Physical Health (3 items).

#### Confirmatory factor analysis (CFA)

**Figure 3:**
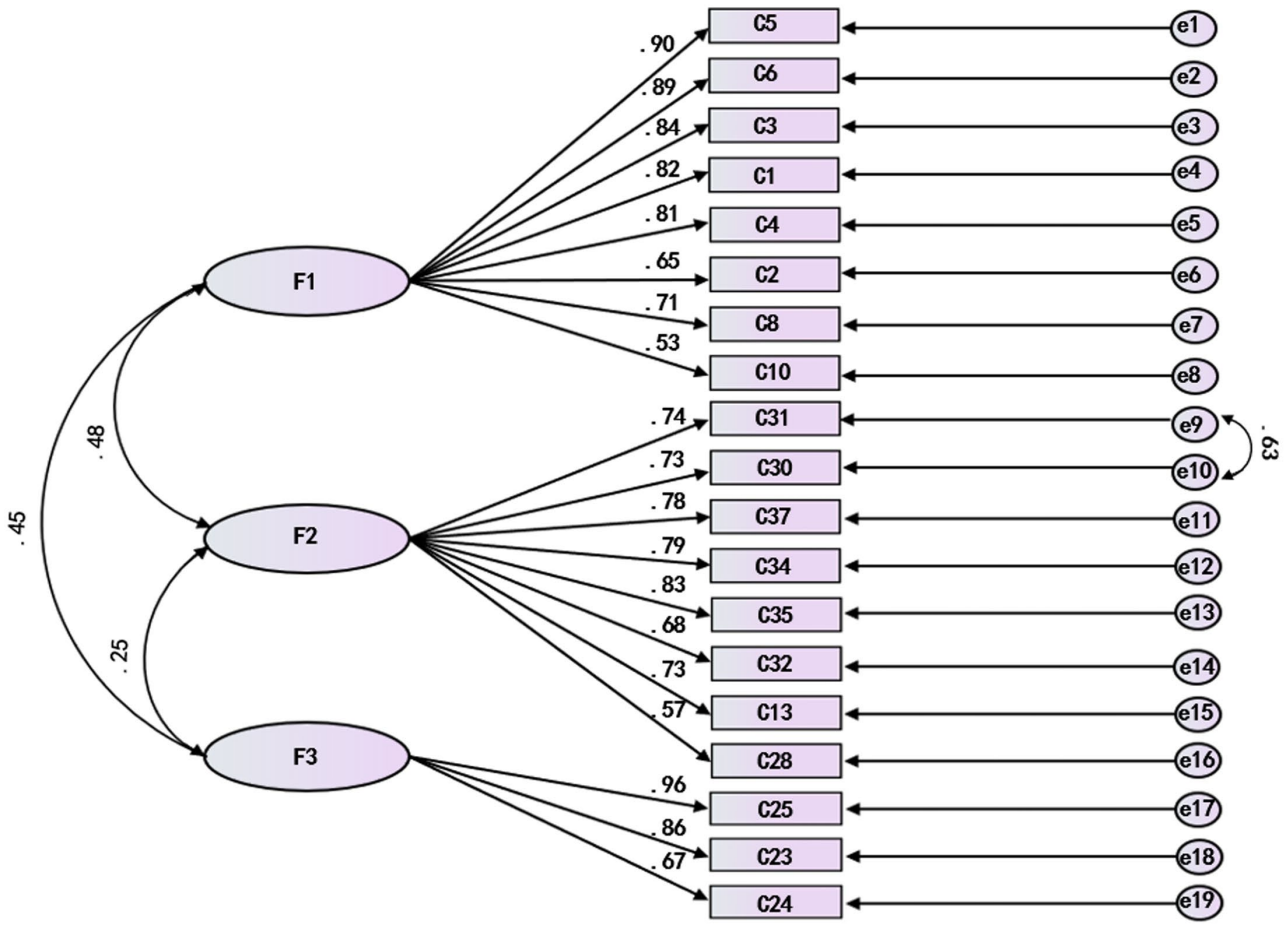
Standardized path coefficients for the confirmatory factor analysis of the 19-item PICS-F scale.

CFA was performed on the second half of the sample (*n =* 224) to test the three-factor, 19-item model derived from EFA. The initial model showed suboptimal fit: *χ*^2^/df = 2.821, RMSEA = 0.090, CFI = 0.904, TLI = 0.890, SRMR = 0.070. Examination of modification indices suggested adding a covariance between the error terms of item C30 (“My leisure and recreational activities have reduced”) and item C31 (“The scope of my social activities has narrowed”). The modification index (MI) was 66.749 and the expected parameter change (EPC) was 0.277. Theoretically, both items reflect the reduction in social and recreational life due to caregiving duties; they represent two related manifestations of the same underlying construct (social withdrawal) rather than independent indicators. After adding this single theoretically justified covariance, model fit improved to adequate levels: *χ*^2^/df = 2.268, RMSEA = 0.075, CFI = 0.934, TLI = 0.924, SRMR = 0.064 (see Table 4). All standardized factor loadings were statistically significant and ranged from 0.572 to 0.962 (Figure 3). The AVE values for the three factors ranged from 0.540 to 0.704, and CR values ranged from 0.875 to 0.922, indicating good convergent validity. The square roots of the AVEs were greater than the inter-factor correlations, supporting discriminant validity (Table 5).

**Table 4:** Model fit indices before and after model modification.

Model	*χ*^2^/df	RMSEA	CFI	TLI	SRMR
Before modification	2.821	0.090	0.904	0.890	0.070
After modification	2.268	0.075	0.934	0.924	0.064
Recommended threshold	<3.000	<0.080	>0.900	>0.900	<0.080

**Table 5:** Discriminant validity: correlations between factors and square roots of AVEs.

Factor	1. Psychological trauma and distress	2. Social and family functioning impairment	3. Deteriorating physical health
1. Psychological Trauma and Distress	0.776		
2. Social and Family Functioning Impairment	0.476	0.735	
3. Deteriorating Physical Health	0.454	0.249	0.839

Diagonal entries (in bold) are the square roots of the Average Variance Extracted (AVE) for each factor. Off-diagonal entries are the inter-factor correlations (all *p* < 0.05). According to Fornell and Larcker (1981), discriminant validity is established when the square root of AVE for each factor is greater than its correlations with other factors.

### Reliability

After the factor structure was established, internal consistency was evaluated using Cronbach’s *α* coefficient for the total scale and each subscale based on the final 19-item, three-factor solution (*n =* 224). The Cronbach’s *α* was 0.918 for the total scale, 0.919 for Factor 1 (Psychological Trauma and Distress, 8 items), 0.903 for Factor 2 (Social and Family Functioning Impairment, 8 items), and 0.851 for Factor 3 (Deteriorating Physical Health, 3 items). The corrected item-total correlations for each subscale were as follows: for Factor1, ranged from 0.512 to 0.844; for Factor2, ranged from 0.550 to 0.758; and for Factor3, ranged from 0.620 to 0.832. The final 19-item PICS-F Assessment Scale is presented in Appendix 4.

## Discussion

This study provided preliminary evidence for a three-factor, 19-item PICS-F Assessment Scale, which demonstrates acceptable preliminary psychometric properties and is tailored to the Chinese cultural context. Further validation with larger and more diverse samples is warranted. The scale comprises three distinct dimensions—Psychological Trauma and Distress, Social and Family Functioning Impairment, and Deteriorating Physical Health, which offers a comprehensive tool to holistically assess the impact of critical illness on family members. This multidimensional assessment is crucial, as intervention and management of PICS-F require a comprehensive, interprofessional approach aimed at addressing multiple symptom clusters simultaneously, including psychological, cognitive, and physiological aspects (Shirasaki et al., 2024).

### General advantages of the PICS-F scale compared with existing instruments

Compared to existing tools commonly used to assess aspects of PICS-F, such as the Hospital Anxiety and Depression Scale (HADS) for psychological distress or the Impact of Event Scale-Revised (IES-R) for post-traumatic stress, the PICS-F Assessment Scale offers distinct advantages. While HADS and IES-R are well-validated for specific symptom domains, they are not designed to capture the full spectrum of PICS-F, particularly the family system impact, social withdrawal, and culturally specific manifestations of distress. This scale integrates these dimensions into a single, concise instrument, reducing assessment burden and providing a more holistic profile of the caregiver’s experience. This comprehensive approach addresses a significant gap in the current toolkit available for PICS-F assessment, especially within Confucian cultural contexts where family dynamics and somatic expressions of distress are prominent.

### Cultural specificity of the scale dimensions

Items C2 (“I constantly want to know the patient’s condition”) and C4 (“I am afraid of answering phone calls from the hospital”) reflect the unique experience of Chinese family caregivers, who are typically deeply involved in communication about the patient’s condition and medical decision-making in ICUs (Liu et al., 2022; Jiang et al., 2025). Item C10 (“I blame myself for not having done enough in the past”) captures the culturally specific manifestation of self-blame rooted in Confucian values of filial piety (xiao) and family responsibility (Peng et al., 2022; Zhang et al., 2024). Items measuring family system disruption C34 (“My care and concern for other family members have decreased”), C35(“Communication among our family members about non-patient matters has decreased”), C37 (“Our family gatherings, outings, and other activities have reduced”), and C13 (“My attention to matters other than the patient has declined”), directly assess the decline in family interaction and cohesion, reflecting “family harmony” as a culturally significant construct in Chinese society (Kavikondala et al., 2016; Zhan and Wang, 2021). Items capturing the economic and occupational consequences of caregiving, such as C32 (“Medical expenses have caused or worsened our family’s financial difficulties”) and C28 (“I am unable to work”), reflect the continuous pathway from catastrophic health expenditure to economic strain and lost developmental opportunities in Chinese families (Li et al., 2023; Tang et al., 2022).

#### Psychological trauma and distress

The Psychological Trauma and Distress dimension captures not only universal symptoms of anxiety and depression but also culturally specific manifestations such as persistent self-blame and somatic expressions of distress. Existing theories have pointed out that cultural norms profoundly influence individuals’ experiences and expressions of trauma (Maercker and Hecker, 2016; Hinton and Lewis‐Fernández, 2011). In the context of Confucian culture, values emphasizing family responsibility and ‘filial piety’ (xiao) may lead family members to internalize the patient’s illness as a personal failure or moral transgression, thereby exhibiting a stronger sense of self-blame rather than outward anger (Kim et al., 2008). Furthermore, psychological distress is often expressed through somatic symptoms or events surrounding the family (such as ‘reduced care for other family members’), which may be more socially acceptable than directly expressing emotions (Ryder et al., 2012). Recent empirical studies focusing on the Chinese population have further confirmed that cultural values are an important factor influencing the manifestation of post-traumatic symptoms (Maercker et al., 2009). Therefore, the dimensions constructed based on qualitative research in this scale may more acutely capture the psychological trauma patterns of Chinese ICU family members in a specific cultural context, which may be overlooked by commonly used Western tools such as HADS and IES-R.

#### Social and family functioning impairment

The Social and Family Functioning Impairment dimension reflects the intertwined impact of caregiving on both social participation and family functioning. It encompasses reduced work efficiency, increased leave, and decreased participation in personal development activities, as well as financial strain, reduced communication, and diminished collective activities within the family. This merged dimension suggests that in the Chinese cultural context, social withdrawal and family crisis are closely intertwined rather than distinct constructs. Our findings align with the qualitative insights from our foundational study (Bao et al., under review), which highlighted how Confucian values like xiao (filial piety) and familial responsibility intensify caregiving burdens in Chinese contexts. Unlike Western studies, where financial strain is often the primary stressor, Chinese caregivers frequently experience profound guilt, self-reproach, and a sense of moral failure when unable to meet familial expectations—a phenomenon deeply rooted in Confucian ethics.

#### Deteriorating physical health

The Deteriorating Physical Health dimension reflects the tangible decline in caregivers’ own health, often neglected due to their focus on the patient. Caregivers often neglect their own health and social roles to fulfill caregiving duties, leading to isolation and physical decline—a pattern exacerbated in cultures where family duties are paramount.

The incidence of PICS-F is relatively high, and it may lead to various issues for patients and their families, seriously affecting the patients’ and their families’ ability to return to normal life (Naaktgeboren et al., 2022; Vester et al., 2022; Alfheim et al., 2019; Avgeri et al., 2023; Shirasaki et al., 2024). Early identification and screening of PICS-F are the foundation for implementing relevant intervention measures (Petrinec and Martin, 2018). Currently, there is a lack of unified diagnostic criteria, and there is no specific assessment tool (Shirasaki et al., 2024). The PICS-F assessment scale developed in this study demonstrates certain validity and reliability. In research, it provides a standardized outcome measure to evaluate the effectiveness of family-centered interventions and to explore the longitudinal trajectory of PICS-F across diverse cultural contexts. Coupled with its relatively short length, it can assist healthcare professionals in screening family members at risk for PICS-F, formulating corresponding intervention measures.

This study demonstrates that the PICS-F assessment scale shows promising preliminary reliability and validity. However, the ultimate value of an assessment tool lies in its ability to improve clinical outcomes. Although interventions aimed at alleviating the burden on family members exist, their effectiveness remains controversial. For instance, a large multi-center RCT conducted by White et al. (2018) revealed that a structured communication intervention led by nurses and centered on family support did not significantly reduce psychological distress among family members (White et al., 2018). This significant finding suggests that universal and undifferentiated intervention strategies may not be sufficient to address the complexity of PICS-F.

So future intervention studies can target these high-risk groups and design more precise support programs and providing timely psychosocial support and culturally sensitive interventions to them (McPeake et al., 2019), thereby enhancing the health status and quality of life of ICU patients and their families.

Based on our findings, we propose the following recommendations for different stakeholders: For healthcare practitioners, the PICS-F Assessment Scale should be utilized for early screening of at-risk family caregivers, enabling the implementation of targeted, culturally sensitive support interventions such as psychological counseling, family meetings, and social resource referrals. For educators, it is crucial to integrate PICS-F awareness and the application of this scale into the training curricula for healthcare students and professionals. Hospital administrators are advised to allocate resources for establishing dedicated family support programs and fostering interdisciplinary collaboration within ICUs. Finally, we urge policy makers to recognize PICS-F as a significant public health issue and to develop national guidelines and support services informed by culturally relevant assessment tools.

### Limitations and future directions

This study has several limitations. First, it was conducted in a single center, which may limit the generalizability of the findings. Second, during the expert consultation stage, the number of experts and their geographical locations were limited. Third, criterion-related validity was not examined. We did not collect measures of related constructs (e.g., caregiver burden, anxiety, and depression) that could have been used to assess convergent validity. We acknowledge this as a major limitation. Future research must include such measures to establish convergent and discriminant validity.

Although we documented the timing of assessment and found that our sample covered a wide range of time points from early ICU admission to post-transfer, the cross-sectional design prevents us from examining within-individual changes over time or establishing causal relationships between time and symptom expression.

Additionally, the sample size for confirmatory factor analysis (*n =* 224) is modest relative to the number of freely estimated parameters in the three-factor 19-item model and does not meet the conservative 10:1 observation-to-parameter guideline suggested by Bentler and Chou (1987). We therefore acknowledge this as a significant limitation. Future validation studies should employ larger samples to confirm the factor structure reported here.

Individual-level patient illness severity data (e.g., APACHE II scores) were not collected in this study. Although we provided contextual information by describing the patient characteristics of each participating ICU in the Participants and Setting section, we were unable to statistically control for illness severity as a confounding variable. This limitation reflects a pragmatic compromise necessitated by the reality that the participating ICUs did not routinely document objective severity scoring systems during the study period. Future studies should incorporate standardized illness severity assessments to more precisely elucidate the relationship between patient clinical status and family PICS-F manifestations. In the future, multi-center large-sample research should be conducted to further validate and apply this scale (Vlake et al., 2022).

## Conclusion

This scale is the first specific instrument designed to assess PICS-F in Chinese family caregivers. Its core contribution is that it achieves, to some extent, a holistic evaluation of psychological, physical, social, and family-level symptoms, thereby overcoming the fragmentation inherent in using multiple generic tools. Its promising preliminary psychometric properties and moderate length suggest potential for routine clinical use, though further validation is required. Once validated, the scale could allow ICU physicians, nurses, and mental health professionals to promptly identify at-risk family members and initiate timely psychological support or interventions. Such proactive care may alleviate family caregivers’ distress and, in turn, positively influence patient recovery. Culturally, the scale is grounded in Confucian values, particularly collectivism and filial piety, and captures culture-specific manifestations such as pronounced family crisis and guilt, which are often overlooked by Western-derived instruments. Future research should validate the scale in multi-center studies across diverse populations and explore its predictive validity and responsiveness to interventions, further establishing its utility in both clinical practice and research.

## Data Availability

The raw data supporting the conclusions of this article will be made available by the authors, without undue reservation.
