# Supplementary material for: Development and preliminary validation of the post-intensive care syndrome-family assessment scale
Source: Front Psychol. 2026 Jun 24;17:1758100. doi: 10.3389/fpsyg.2026.1758100 (PMC13341686; doi:10.3389/fpsyg.2026.1758100)
Supplement: Supplementary file 1 [file Supplementary_file_1.docx]

Appendix 1 Dimensions and Items of the Preliminary PICS-F Item Pool

| Dimension | Item |
| --- | --- |
| Psychological Trauma and Distress | Feeling nervous or anxious |
|  | Feeling the wait is agonizing |
|  | Being afraid of answering phone calls the hospital |
|  | Feeling depressed |
|  | Being unable to control the urge to cry tear up |
|  | Feeling sad |
|  | Temper worsening |
|  | Irritability |
|  | Feeling helpless |
|  | Being prone to random negative thoughts |
|  | Blaming oneself for not having done enough in the past |
|  | Losing interest in things previously enjoyed |
|  | Narrowing of attention span |
|  | Difficulty concentrating |
|  | Memory decline |
|  | Slowed thinking |
|  | Feeling afraid when recalling critical illness memories |
|  | Involuntary recurrence of critical illness memories |
|  | The ICU environment, patients, and other family members triggering painful critical illness memories |
|  | Deliberately avoiding critical illness memories |
|  | Feeling psychologically traumatized |
|  | Deliberately avoiding things that used to make one happy |
|  | Only feeling at ease when staying at the hospital or ICU entrance |
|  | Withdrawing into oneself |
|  | Decreased motivation |
| Deteriorating Physical Health | Decreased sleep quality (e.g., difficulty falling asleep, frequent dreams or nightmares, easily awakened, reduced sleep duration) |
|  | Poor appetite |
|  | Weight loss |
|  | Fatigue |
|  | Development of new health problems |
|  | Simplification of diet structure |
| Social Withdrawal | Being unable to work or work being affected |
|  | Being forced to reduce social activities |
|  | Unwillingness to participate in social activities |
|  | Narrowing of daily activity areas or scope |
|  | One’s own life or activities revolving entirely around the patient |
| Family Crisis | Family financial hardship |
|  | Family conflicts arising from treatment plans or financial burden |
|  | Decreased care and concern for other family members (e.g., elderly, children) due to accompanying or caring for the patient |
|  | Reduction in family collective activities |
|  | Family atmosphere becoming sorrowful and oppressive |
|  | Income being affected due to accompanying the patient for medical care or providing care |

Note: PICS-F = Post-Intensive Care Syndrome-Family.
