# Supplementary material for: Development and preliminary validation of the post-intensive care syndrome-family assessment scale
Source: Front Psychol. 2026 Jun 24;17:1758100. doi: 10.3389/fpsyg.2026.1758100 (PMC13341686; doi:10.3389/fpsyg.2026.1758100)
Supplement: Supplementary file 2 [file Supplementary_file_2.docx]

Appendix 2 Revision of Items Across All Dimensions After Expert Consensus Meeting

| Original Item | Revised Item  (with Subject and Context) | Rationale for Revision |
| --- | --- | --- |
| Dimension 1: Psychological Trauma and Distress | Psychological trauma and distress (impact on emotional, cognitive, and other related psychological domains in family members) | Added dimension definition. |
| Feeling nervous or anxious | I feel tense and uneasy. | Add the subject “I” and further clarify the meaning of the sentence. |
| Feeling the wait is agonizing | I constantly want to know the patient’s condition. | Reframed an internal feeling into a more specific, observable behavior. |
| Being afraid of answering phone calls the hospital | I am afraid of answering phone calls from the hospital. | Added subject “I” and specified context "from the hospital". |
| Feeling depressed | I feel depressed. | Added subject “I” for clarity. |
| Being unable to control the urge to cry tear up | I can’t help but want to cry or shed tears. | Added subject “I” for clarity. |
| Feeling sad | (Deleted) | Duplicate with “Feeling depressed” and “Being unable to control the urge to cry tear up”. |
| Temper worsening | I get angry or lose my temper easily. | Combined with “Irritability” and rephrased into more common, non-overlapping language. |
| Irritability | (Merged with "Worsened temper" above) | Merged due to conceptual overlap; term “Irritability” is considered too technical for a layperson scale. |
| Feeling helpless | I feel helpless. | Added subject “I” for clarity. |
| Being prone to random negative thoughts | I tend to overthink when I have free time. | Added subject “I” and context “when I have free time” to specify the trigger. |
| Blaming oneself for not having done enough in the past | I blame myself for not having done enough in the past. | Added subject “I” for clarity. |
| Losing interest in things previously enjoyed | I have lost interest in things I used to enjoy. | Added subject “I” for clarity and personalization. |
| Narrowing of attention span | My focus has narrowed, and I pay less attention to things unrelated to the patient. | Added subject “I” and specified the context to clarify the manifestation. |
| Difficulty concentrating | I have difficulty concentrating. | Added subject “I” for clarity. |
| Memory decline | My memory has worsened. | Added subject “My” and rephrased using more colloquial language. |
| Slowed thinking | My mind feels slow. | Added subject “My” and rephrased using more colloquial language. |
| Feeling afraid when recalling critical illness memories | When recalling scenes and memories of patients in critical condition, I feel fear and distress. | Added subject “I” and define the meaning of critical memory with plain expressions. |
| Involuntary recurrence of critical illness memories | Visions or memories of the patient's critical condition pop into my mind unexpectedly. | “Involuntary recurrence of critical illness memories” and “The ICU environment, patients, and other family members triggering painful critical illness memories” both refer to flashback; Merge them into a single item, define the implication of critical illness memory, add the subject and use more accessible wording. |
| The ICU environment, patients, and other family members triggering painful critical illness memories |  |  |
| Deliberately avoiding critical illness memories | (Deleted) | Classified as avoidant coping behavior rather than manifestation. |
| Feeling psychologically traumatized | (Deleted) | Deleted as the description was vague, overly broad, and overlapped with other items. |
| Deliberately avoiding things that used to make one happy | (Deleted) | Deleted as it represents avoidant coping behavior rather than a core cognitive symptom. |
| Only feeling at ease when staying at the hospital or ICU entrance | (Deleted) | Classified as avoidant coping behavior rather than manifestation. |
| Withdrawing into oneself | I don’t feel like interacting with others. | Added subject "I" for clarity and rephrased using more colloquial language. |
| Decreased motivation | (Deleted) | It pertains to behavior rather than cognition. |
| (New item supplemented in accordance with qualitative interview data and expert advice) | I suspect that I have also fallen ill. | Relatives reported persistent excessive worry over the patient’s treatment and occasional self-perceived illness, leading to the addition of this item. Add this item according to qualitative interview data and expert advice. |
| Dimension 2: Deteriorating Physical Health | Deteriorating physical health (physical health issues in family members) | Added dimension definition and modified the description. |
| Decreased sleep quality (e.g., difficulty falling asleep, frequent dreams or nightmares, easily awakened, reduced sleep duration) | My sleep quality has decreased (e.g., difficulty falling asleep, more dreams/nightmares, waking up easily, sleeping less). | Added subject “My” for clarity. |
| Poor appetite | I have little to no appetite. | Added subject “I” and rephrased using more colloquial language. |
| Weight loss | I have lost weight. | Added subject “I” for clarity. |
| Fatigue | I feel tired easily. | Added subject “I” and rephrased using more colloquial language. |
| Development of health problems | My pre-existing illnesses have relapsed or worsened. | Term too broad/vague. Item split into three specific entries based on qualitative feedback from family members to accurately capture different manifestations (relapse, new issues, self-medication/help-seeking). |
|  | I have developed new physical discomfort or illnesses. |  |
|  | I have started taking medication or seeking medical care for myself. |  |
| Simplification of diet structure | (Deleted) | Deleted as it represents a coping behavior rather than a direct symptom of health deterioration. |
| Dimension 3:  Social Withdrawal | Social Withdrawal (impact on work, social activities, and other related domains) | Added dimension definition. |
| Being unable to work or work being affected | My work efficiency has decreased. | The original term was overly broad and non-specific. Based on qualitative descriptions, it was subdivided into four distinct items to capture varying degrees of impact: reduced efficiency, increased absenteeism, reduced self-improvement, and work incapacity. |
|  | I have taken more frequent or longer leave from work. |  |
|  | My participation in self-improvement activities (e.g., training, courses) has decreased. |  |
|  | I am unable to work. |  |
| Being forced to reduce social activities | My recreational activities have decreased. | Both items described impacts on social activities. They were replaced with two newly formulated items based on qualitative feedback to clearly distinguish between reductions in recreational activities and hobby-related activities. |
| Unwillingness to participate in social activities | My hobby-related activities have decreased. |  |
| Narrowing of daily activity areas or scope | My daily activity range has become smaller. | Added subject “My” and simplified the description. |
| One’s own life or activities revolving entirely around the patient | (Deleted) | The connotation was unclear. Upon tracing family descriptions, this was identified as a reason for social shrinkage, not a measurable manifestation itself, and was therefore deleted. |
| Dimension 4:  Family crisis | Family crisis (impact on economic, emotional, and other related domains) | Dimension Description. |
| Family financial hardship | High expenses lead to or exacerbate household financial hardship. | The original term was overly broad. Based on qualitative descriptions, it was subdivided into two specific items: increased expenses and reduced income. |
|  | Household and personal income is adversely affected. |  |
| Family conflicts arising from treatment plans or financial burden | Family conflicts have arisen regarding the patient's treatment, care, or expenses. | Rephrased using more colloquial and direct language. |
| Decreased care and concern for other family members (e.g., elderly, children) due to accompanying or caring for the patient | My care and concern for other family members have decreased. | Added subject “My” and simplified the description. |
| Reduction in family collective activities | Our family gatherings or outings have decreased. | The original term was overly broad. Specified the context to common family activities. |
| Family atmosphere becoming sorrowful and oppressive | I feel more depressed and distressed after returning home. | Added subject “I”. |
| Income being affected due to accompanying the patient for medical care or providing care | (Deleted) | Duplicates the content of “Family financial hardship”. |
| (New item supplemented in accordance with qualitative interview data and expert advice) | Communication between family members has decreased. | Family members avoid communicating beyond patient-related matters. Add this item according to qualitative interview data and expert advice. |
| (New item supplemented in accordance with qualitative interview data and expert advice) | Other family members have fallen ill due to accompanying the patient for medical care or providing care. | Other family members were severely affected, even to the point of becoming ill. Add this item according to qualitative interview data and expert advice. |
