# Supplementary material for: Development and preliminary validation of the post-intensive care syndrome-family assessment scale
Source: Front Psychol. 2026 Jun 24;17:1758100. doi: 10.3389/fpsyg.2026.1758100 (PMC13341686; doi:10.3389/fpsyg.2026.1758100)
Supplement: Supplementary file 3 [file Supplementary_file_3.docx]

Appendix 3 Post-Intensive Care Syndrome-Family (PICS-F) Assessment Questionnaire (Validation Version)

**Post-Intensive Care Syndrome-Family (PICS-F) Assessment Questionnaire**

Dear Family Member of an ICU Patient,

We are healthcare professionals from the First Affiliated Hospital of Kunming Medical University. We are currently conducting a survey on "Post-Intensive Care Syndrome-Family (PICS-F)." This research aims to understand the impact of a patient's critical illness and ICU admission on their family members, with the goal of facilitating better medical and nursing support for both ICU patients and their families.

This study adheres to ethical principles. Your participation is entirely voluntary, and you may withdraw at any time. All information you provide will be used solely for research purposes and will be kept strictly confidential.

The questionnaire consists of three main sections:

1.Patient Demographic Information Sheet

2.Family Member Demographic Information Sheet

3.Post-Intensive Care Syndrome-Family (PICS-F) Assessment Scale

Your feedback is crucial to the success of our research. We sincerely thank you for your time and support.

Instructions for Completion:

[1] There are no right or wrong answers. Please respond based on your personal experiences and feelings.

[2] For multiple-choice questions, please select only one answer by marking "√" in the box next to your chosen option.

[3] For open-ended questions, please write your answer directly on the "_____" line provided.

A、Patient Demographic Information Sheet

1、Patient’s Sex:

□Male □Female

2、Patient's Age: ________ years old

3、Patient's ICU Length of Stay: _________ days

4、Days since ICU Discharge: _________ days

5、Reason for ICU Admission:_______________________________________

6、Route of ICU Admission:
□ Emergency Department □ General Ward □ Post-operation □ Transfer from another hospital

7、Patient's Medical Payment Type:

□ Resident Basic Medical Insurance □ Employee Basic Medical Insurance □ Out-of-pocket

□ Medical Assistance for low-income families □ Commercial Health Insurance

□ Other: ____________

B、Family Member Demographic Information Sheet

1、Your relationship to the patient:
□ Parent □ Spouse □ Sibling □ Child

2、Your Sex:
□ Male □ Female

3、Your Age: _________ years old

4、Your Ethnicity:

□ Han Chinese □ Ethnic Minority

5、Your Educational Level:

□ Primary school or below □ Junior high school □ High school / Vocational school

□ College / Bachelor's degree □ Master's degree or above

6、Your Occupation:

□ Government/Institutional employee □ Corporate employee □ Self-employed/Business

□ Farmer □ Freelancer □ Unemployed □ Retired

□ Other: ______________

7、Your total household monthly income falls within which range?

□ 0–3000 CNY □ 3001–6000 CNY □ 6001–9000 CNY □ >9000 CNY □ Unknown

8、How well do you understand the patient's condition?

□ Very poor □ Poor □ Moderate □ Good

C、Post-Intensive Care Syndrome-Family (PICS-F) Assessment Scale

Instructions: Since the patient's ICU admission until now, to what extent have the patient's condition, treatment, care needs, expenses, etc., caused the following impacts on you? Please indicate your response based on your actual situation by marking "√" in the appropriate box for each statement.

| No. | Item | Strongly Disagree | Disagree | Neutral | Agree | Strongly Agree |
| --- | --- | --- | --- | --- | --- | --- |
| 1 | I feel tense and uneasy. | □ | □ | □ | □ | □ |
| 2 | I constantly want to know the patient's condition. | □ | □ | □ | □ | □ |
| 3 | I feel helpless. | □ | □ | □ | □ | □ |
| 4 | I am afraid of answering phone calls from the hospital. | □ | □ | □ | □ | □ |
| 5 | I feel depressed. | □ | □ | □ | □ | □ |
| 6 | I can’t help but want to cry or shed tears. | □ | □ | □ | □ | □ |
| 7 | I get angry or lose my temper easily. | □ | □ | □ | □ | □ |
| 8 | I tend to overthink when I have free time. | □ | □ | □ | □ | □ |
| 9 | I suspect that I have also fallen ill. | □ | □ | □ | □ | □ |
| 10 | I blame myself for not having done enough in the past. | □ | □ | □ | □ | □ |
| 11 | I don’t feel like interacting with others. | □ | □ | □ | □ | □ |
| 12 | I have lost interest in things I used to enjoy. | □ | □ | □ | □ | □ |
| 13 | My attention to matters other than the patient has declined. | □ | □ | □ | □ | □ |
| 14 | I have difficulty concentrating. | □ | □ | □ | □ | □ |
| 15 | My memory has worsened. | □ | □ | □ | □ | □ |
| 16 | My mind feels slow. | □ | □ | □ | □ | □ |
| 17 | Visions or memories of the patient's critical condition pop into my mind unexpectedly. | □ | □ | □ | □ | □ |
| 18 | When recalling scenes and memories of patients in critical condition, I feel fear and distress. | □ | □ | □ | □ | □ |
| 19 | My sleep quality has decreased (e.g., difficulty falling asleep, more dreams/nightmares, waking up easily, sleeping less). | □ | □ | □ | □ | □ |
| 20 | I have little to no appetite. | □ | □ | □ | □ | □ |
| 21 | I have lost weight. | □ | □ | □ | □ | □ |
| 22 | I feel tired easily. | □ | □ | □ | □ | □ |
| 23 | My pre-existing illnesses have relapsed or worsened. | □ | □ | □ | □ | □ |
| 24 | I have developed new physical discomfort or illnesses. | □ | □ | □ | □ | □ |
| 25 | I have started taking medication or seeking medical care for myself. | □ | □ | □ | □ | □ |
| 26 | My work efficiency has decreased. | □ | □ | □ | □ | □ |
| 27 | I have taken more frequent or longer leave from work. | □ | □ | □ | □ | □ |
| 28 | I am unable to work. | □ | □ | □ | □ | □ |
| 29 | My participation in self-improvement activities (e.g., training, courses) has decreased. | □ | □ | □ | □ | □ |
| 30 | My recreational activities have decreased. | □ | □ | □ | □ | □ |
| 31 | The scope of my social activities has decreased. | □ | □ | □ | □ | □ |
| 32 | Medical expenses have caused or worsened our family's financial difficulties. | □ | □ | □ | □ | □ |
| 33 | Our family income has been impacted. | □ | □ | □ | □ | □ |
| 34 | My care and concern for other family members have decreased. | □ | □ | □ | □ | □ |
| 35 | Communication among our family members about non-patient matters has decreased. | □ | □ | □ | □ | □ |
| 36 | I feel more depressed and distressed after returning home. | □ | □ | □ | □ | □ |
| 37 | Our family gatherings, outings, and other activities have reduced. | □ | □ | □ | □ | □ |
| 38 | Other family members have fallen ill due to accompanying the patient for medical care or providing care. | □ | □ | □ | □ | □ |
| 39 | Family conflicts have arisen regarding the patient's treatment, care, or expenses. | □ | □ | □ | □ | □ |
