# Supplementary material for: Development and preliminary validation of the post-intensive care syndrome-family assessment scale
Source: Front Psychol. 2026 Jun 24;17:1758100. doi: 10.3389/fpsyg.2026.1758100 (PMC13341686; doi:10.3389/fpsyg.2026.1758100)
Supplement: Supplementary file 4 [file Supplementary_file_4.docx]

Appendix 4 Post-Intensive Care Syndrome-Family (PICS-F) Assessment Questionnaire (Final Version)

| No. | Item | Strongly Disagree | Disagree | Neutral | Agree | Strongly Agree |
| --- | --- | --- | --- | --- | --- | --- |
| Factor 1–Psychological Trauma and Distress (8 items) | | | | | | |
| 1 | I feel depressed. | □ | □ | □ | □ | □ |
| 2 | I can’t help but want to cry or shed tears. | □ | □ | □ | □ | □ |
| 3 | I feel helpless. | □ | □ | □ | □ | □ |
| 4 | I feel tense and uneasy. |  |  |  |  |  |
| 5 | I am afraid of answering phone calls from the hospital. | □ | □ | □ | □ | □ |
| 6 | I constantly want to know the patient's condition. | □ | □ | □ | □ | □ |
| 7 | I tend to overthink when I have free time. | □ | □ | □ | □ | □ |
| 8 | I blame myself for not having done enough in the past. | □ | □ | □ | □ | □ |
| Factor 2–Social and Family Functioning Impairment (8 items) | | | | | | |
| 9 | The scope of my social activities has decreased. | □ | □ | □ | □ | □ |
| 10 | My recreational activities have decreased. | □ | □ | □ | □ | □ |
| 11 | Our family gatherings, outings, and other activities have reduced. | □ | □ | □ | □ | □ |
| 12 | My care and concern for other family members have decreased. | □ | □ | □ | □ | □ |
| 13 | Communication among our family members about non-patient matters has decreased. | □ | □ | □ | □ | □ |
| 14 | Medical expenses have caused or worsened our family's financial difficulties. | □ | □ | □ | □ | □ |
| 15 | My attention to matters other than the patient has declined. | □ | □ | □ | □ | □ |
| 16 | I am unable to work. | □ | □ | □ | □ | □ |
| Factor 3–Deteriorating Physical Health (3 items) | | | | | | |
| 17 | I have started taking medication or seeking medical care for myself. | □ | □ | □ | □ | □ |
| 18 | My pre-existing illnesses have relapsed or worsened. | □ | □ | □ | □ | □ |
| 19 | I have developed new physical discomfort or illnesses. | □ | □ | □ | □ | □ |

Note: Each item is scored from 1 (Strongly Disagree) to 5 (Strongly Agree).
